# Effects of Rubber Core on the Mechanical Behaviour of the Carbon–Aramid Composite Materials Subjected to Low-Velocity Impact Loading Considering Water Absorption

**DOI:** 10.3390/ma17164055

**Published:** 2024-08-15

**Authors:** Stefania Ursache, Camelia Cerbu, Anton Hadăr, Horia Alexandru Petrescu

**Affiliations:** 1 Department of Mechanical Engineering, Faculty of Mechanical Engineering, Transilvania University of Brasov, B-dul Eroilor, No. 29, 500036 Brasov, Romania; stefania.olareanu@unitbv.ro; 2 Department of Strength of Materials, Faculty of Industrial Engineering and Robotics, National University of Science and Technology Politehnica Bucharest, 313 Splaiul Independentei, 060042 Bucharest, Romania; anton.hadar@upb.ro (A.H.); horia.petrescu@upb.ro (H.A.P.); 3 Academy of Romanian Scientists, Ilfov Street No. 3, Sector 5, 050044 Bucharest, Romania; 4 Technical Sciences Academy of Romania, Dacia Boulevard No. 26, Sector 1, 010413 Bucharest, Romania

**Keywords:** impact, low velocity, hybrid composite material, carbon, aramid, water absorption

## Abstract

The large-scale use of composite materials reinforced with carbon–aramid hybrid fabric in various outdoor applications, which ensures increased mechanical resistance including in impact loadings, led to the need to investigate the effects of aggressive environmental factors (moisture absorption, temperature, thermal cycles, ultra-violet rays) on the variation of their mechanical properties. Since the literature is still lacking in research on this topic, this article aims to compare the low-velocity impact behaviour of two carbon–aramid hybrid composite materials (with and without rubber core) and to investigate the effects of water absorption on impact properties. The main objectives of this research were as follows: (i) the investigation of the mechanical behavior in tests for two impact energies of 25 J and 50 J; (ii) comparison of the results obtained in terms of the force, displacement, velocity, and energy related to the time; (iii) analysis of the water absorption data; (iii) low-velocity impact testing of wet specimens after saturation; (iv) comparison between the impact behaviour of the wet specimens with that of the dried ones. One of the main findings was that for the wet specimens without rubber core, absorbed impact energy was 16% less than the one recorded for dried specimens at an impact energy of 50 J. The failure modes of the dried specimens without rubber core are breakage for both carbon and aramid fibres, matrix cracks, and delamination at matrix–fibre interfaces. The degradation for the wet specimens with rubber core is much more pronounced because the decrease in the absorbed impact energy was 53.26% after 10,513 h of immersion in water and all the layers were broken.

## 1. Introduction

Currently, there is a significant interest regarding the use of hybrid composite materials for applications in automotive, civil engineering, aerospace, marine or offshore industries and ground transportation [1,2,3,4,5].

By using multiple types of fibres to strengthen the composite material, a unique material is achieved that combines the benefits of each individual component while also minimising their undesirable characteristics. Considering these advantages, hybrid composite materials have gained popularity in the last two decades due to their mechanical properties and low weight [6,7,8,9]. There are hybrid composite materials reinforced with two types of synthetic and/or natural fibres (carbon and aramid fibres; glass fibres and flax or jute fibres) [10,11,12] or reinforced only with natural fibres (vegetable fibres with wood fibres; several types of wood fibres), the latter being used for thermal or acoustic protection [13,14].

Carbon–aramid hybrid composites are an excellent example of high-performance properties, specifically in terms of their impressive strength–weight and stiffness–weight ratios. This makes them highly suitable for load-bearing applications in the automotive and aerospace industries. Due to their lightweight nature, these composites can withstand low-velocity impact loadings that may occur during various operational, manufacturing, and maintenance processes [8,9,15,16]. Epoxy resin-based composite materials reinforced with carbon fibres have gained significant recognition as a highly promising category of structural materials, due to their exceptional static strength, stiffness properties, and resistance to corrosion [15,17,18,19,20]. There are even studies that have studied the low-speed impact behaviour of composite materials reinforced with carbon fibres, even if carbon fibres, although they have high resistance, are less flexible than aramid fibres [21,22]. The composite materials reinforced with aramid fibres have been widely used in ballistic applications and as lightweight armour structures and protective helmets for military equipment and for civil engineering, due to their high impact strength [23,24,25]. For example, there are many applications in civil engineering (ballistic protection panels, highway parapets, protective helmets) for which the composite structures are subjected to impact loadings. Therefore, currently, the study of the impact response on composite materials has become a research area of great interest, especially for hybrid configurations engineering applications, in civil engineering [26,27,28].

Based on recent research studies, it can be concluded that the combination of carbon fibres with aramid fibres enhances the mechanical performance of the hybrid composite reinforced with both fibres under impact loading conditions, while also mitigating the extent of strength deterioration after impact, as opposed to the reinforced carbon epoxy composites [9,27,29]. Jang et al. [30] have focused on the comparison of the impact response between carbon fibre-reinforced composites, aramid fibre-reinforced composites, and carbon–aramid hybrid composites in order to analyse the influence of aramid fibres on the composite structure. Gustin et al. [27] also focused on the improvement of the impact properties by replacing the carbon fibre reinforcement material with aramid fibres for the impact side face sheet layers.

The authors of this paper have already investigated the mechanical and elastic properties (including Poisson’s ratio) of the composite materials reinforced with carbon–aramid fabric in tensile and bending tests using an additional digital image correlation method [10].

Taking into account the sensitivity of the composite materials reinforced with aramid fibres regarding the effects of moisture absorption [31,32,33,34,35], it had become a necessity to study the effects of moisture absorption on the mechanical properties of the hybrid composites reinforced with both carbon and aramid fibres [36,37,38].

The impact behaviour of the carbon–aramid hybrid composites with different stacking of configurations had been investigated over the years, by several authors and it was noticed the positive effects in impact response and energy absorption for these structures by adding aramid fibres. The water absorption behaviours and impact properties of hybrid composites that contain aramid fibres were also evaluated in different studies and the experimental results revealed that water absorption affects the impact strength of the composite materials [37,39,40,41]. A great part of these studies was performed considering multi-layered hybridisation with plies reinforced either with carbon fibres or with aramid fibres for particular applications [27,30,42]. Regarding the evaluation of the effects of the temperature or moisture absorption on the hybrid composites containing layers reinforced either with carbon fibres or with aramid fibres, it can be noted that in recent years a real interest has been shown among researchers in the investigation of the degradation of the mechanical properties of such composites [8,43,44,45,46,47]. But, the literature lacks research on the long-term effects of water absorption for composite materials reinforced with carbon–aramid hybrid fabrics.

Considering the previous short description of the state-of-the-art of the scientific works published, the conclusion is that the low-velocity impact response needs further investigation for the composite materials reinforced with hybrid carbon–aramid fabric. The response of such hybrid carbon–aramid composites tested after long-term water absorption is also required considering the impact protection applications of composite materials reinforced with aramid and carbon fibres, in civil engineering and transports (strengthening wooden or concrete beams, impact protection parapets) [31]. Therefore, the main purpose of this study is to investigate the low-velocity impact response of two types of hybrid composite plates based on epoxy resin reinforced with carbon–aramid fabric: a composite plate whose eight layers are reinforced with hybrid carbon–aramid fabric; a sandwich composite material having a rubber core and two face sheets containing three layers reinforced with the same carbon–aramid fabric.

For this purpose, the main objectives of this research are (i) low-velocity drop impact testing for both types of hybrid composite plates reinforced with carbon–aramid woven fabric at two levels of the impact energies (25 J and 50 J); (ii) the comparison of the results obtained for the two types of plates (with and without a rubber core) in terms of the force, displacement, and energy related to the time; (iii) the analysis of the absorbed water content as a function of time, until saturation (after, for the composites involved; (iv) the comparison of the impact behaviour of the wet samples compared to the dried ones in terms of the absorbed impact energy, the deformation, the rebound speed, and the breaking modes. In order to report the failure modes that occurred during the low-velocity impact tests, the microscopic analyses of the specimens after the impact tests were performed.

For both types of carbon–aramid hybrid composite materials (with and without a rubber core), the low-velocity impact testing was carried out taking into account two levels of the impact energy, 25 J and 50 J. The tests at the impact energy of 25 J were necessary to be able to record the amount of the failure energy absorbed in impact tests in case the composite plates would have been perforated in the impact tests at the energy of 50 J because the degradation of the composites after the immersion in water until saturation. The time of immersion until saturation was considered after stabilising the amount of water absorbed by the tested composite materials.

## 2. Materials and Methods

### 2.1. Materials

The carbon–aramid hybrid woven fabric used in this study is the SIGRATEX H W215-TW2/2 type manufactured by SGL Carbon in Wiesbaden, Germany. This fabric consists of two intersecting yarn networks—a warp direction and a weft direction—both containing carbon fibre yarns as well as aramid fibre yarns.

The main material characteristics of the carbon–aramid hybrid woven fabric are shown in Table 1 [48].

Epoxy resin Epolam 2031 (Axon Technologies, Eaton, Rapids, MI, USA) was mixed with its corresponding Epolam 2031 hardener (Axon Technologies, Eaton, Rapids, MI, USA) before the impregnation of the carbon–aramid hybrid woven fabric, with a mix ratio by volume being equal to 100:33 according to the datasheet of the Epolam 2031 epoxy resin [49]. The physical and mechanical properties of the Epolam 2031 complex (epoxy resin and hardener) used in this research are given in Table 2 [49].

Two sets of specimens were manufactured for the low-velocity impact testing. All the specimens were square panels having the dimensions 70 mm × 70 mm. The first set of samples was cut from a composite panel reinforced just with carbon–aramid hybrid fabric, which has eight layers, and was manufactured by hand-layup technology, resulting in an average thickness of approximately 2.6 mm. After moulding, the composite panel was kept at room temperature (20 °C ± 2 °C) for two weeks, before cutting the specimens by using the cutting machine with a water jet.

The second set of specimens was cut from a sandwich composite panel, having both upper and bottom faces made of three layers of composite material reinforced with carbon–aramid hybrid fabric and a rubber core whose thickness is 2 mm [50]. The rubber core is a rubber sheet manufactured by ADEGO S.A. (Târgu Jiu, Romania) having a hardness of 60–75 Sh A and a strain at break of 150% [50]. The same technology described above was used, resulting in an average sample thickness of approximately 3.75 mm.

The fibre content was equal to 45 wt.% both for carbon–aramid composites without rubber core and for the sheet faces of the sandwich composite.

The same technology described above was used resulting in an average sample thickness of approximately 3.75 mm. The faces of the sandwich plates were glued to the rubber core using the Bison 10431 two-component epoxy adhesive [51]. Finally, 20 impact specimens were cut from each type of composite panel for the low-velocity impact testing. From the same batch of specimens, ten specimens of each set were completely immersed in water and held until saturation, in order to investigate the effect of water absorption on their behaviour at low-velocity impact. Table 3 summarises all the composite material samples prepared for the low-velocity impact tests at two different energies (25 J and 50 J impact tests) showing their material structure. The difference between the two composite materials tested is that two layers reinforced with aramid–carbon hybrid fabric were replaced with the rubber core in the sandwich composite materials in order to comparatively analyse their behaviour in impact tests before water immersion and after saturation.

**Figure 1:**
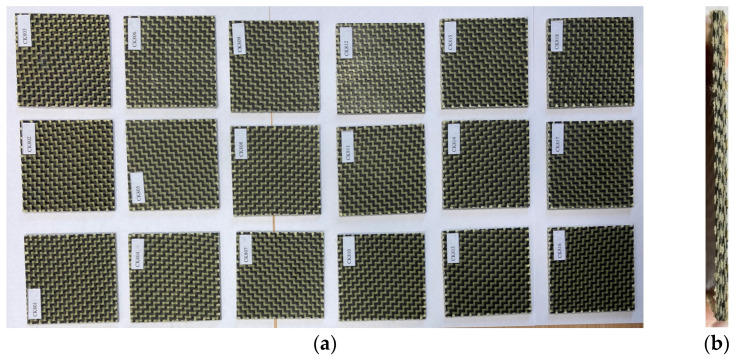
Impact specimens CK made of eight layers reinforced with carbon–aramid hybrid fabric: (**a**) front view; (**b**) cross-section view.

**Figure 2:**
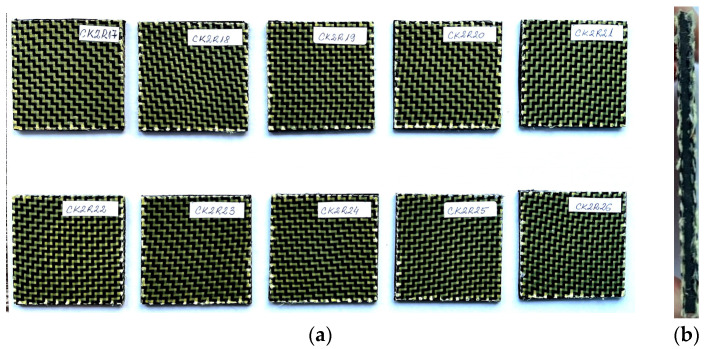
Sandwich specimens CKR having both faces made of three layers reinforced with carbon–aramid hybrid fabric and a rubber core: (**a**) front view; (**b**) cross-section view.

Figure 1 and Figure 2 present the photos of the dried specimens before impact tests.

### 2.2. Experimental Method

#### 2.2.1. Low-Velocity Drop Impact Test

**Figure 3:**
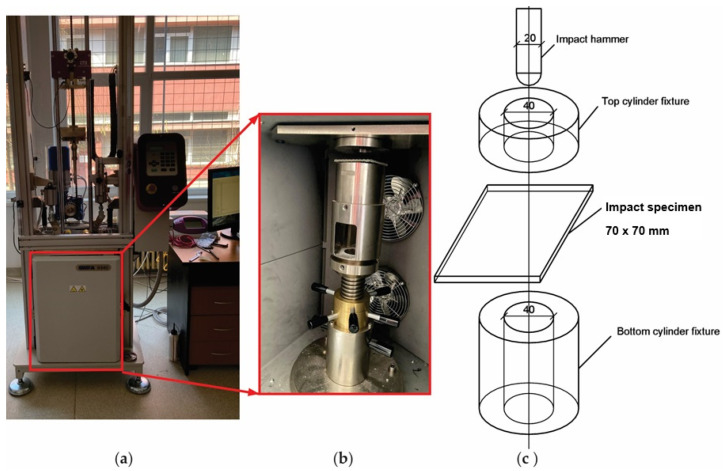
Low-velocity impact test setup: (**a**) photo of Instron CEAST 9340 drop weight impact machine; (**b**) photo of the fixture device of the specimen; (**c**) scheme with dimensions for the impact specimen and for the devices of the impact machine.

The low-velocity drop impact tests were carried out by the Instron CEAST 9340 impact machine (Norwood, MA, USA) shown in Figure 3 at room temperature (20 °C ± 2 °C). The testing system consists of an impact hammer equipped with a force cell (maximum 22 kN), clamping fixtures, and a data acquisition system (Figure 3a). The main technical characteristics of the Instron CEAST 9340 machine are maximum impact energy of 405 J; maximum impact velocity of 4.65 m/s; a mass of the drop weight in the range between 1 kg and 37.5 kg; and drop height in the range between 0.03 m and 1.10 m. It is well known that the greater the velocity or mass, the higher the impact energy. The impact machine is digitally controlled by the CEAST VisualIMPACT V.6 software. The variations in the impact force, displacement, and elastic strain energy related to the time, which are used to evaluate the effects of the impact test, were recorded in the software of the impact machine by the data acquisition system of that equipment. The frequency of the data acquisition was set to every 0.002 ms before each low-velocity impact test.

The photo of the Instron CEAST 9340 impact machine is shown in Figure 3a while the photo of the fixture devices of the specimen during the impact test is shown in Figure 3b. Each square impact specimen tested, whose dimensions are 70 mm × 70 mm, was firmly fixed in the testing machine by placing each plate specimen on a hollow cylinder support having an internal diameter of 40 mm (Figure 3c). The testing procedure is similar to the one presented in another research [52] by two of the co-authors of this paper. A fastener acted by two hydraulic cylinders fix the specimen through a steel ring having the same inner diameter of 40 mm as the support cylinder (Figure 3c).

The impact specimens, whose dimensions are 70 mm × 70 mm, were cut from the hybrid carbon–aramid composite panel, by using the cutting machine with a water jet. As has been mentioned before, two sets containing initially six specimens were prepared for low-velocity drop impact test at two levels of impact energies of 25 J and 50 J, respectively. Just results for five specimens were considered in this research because one specimen of each set was used just for test calibration.

In order to control the energy level for our study, the impact tests were performed with the following configurations: (i) for 50 J energy impact level, the impact hammer weight was 10.728 kg and the start velocity was 3.05 m/s; (ii) for 25 J energy impact level, the impact hammer weight was 10.728 kg and the start velocity was 2.16 m/s. For each impact energy, the results were analysed for each sample. According to the standards ISO 6603-2 [53] and ISO 7765-2 [54], the diameter of the impact hammer in both cases was 20 mm. The work method previously described was used both for the dried specimens (before immersion in water) and for the wet specimens (after immersion in water until saturation).

#### 2.2.2. Moisture Absorption

**Figure 4:**
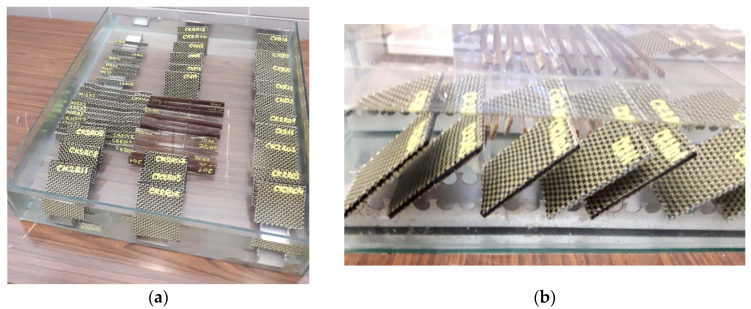
Impact specimens immersed in water: (**a**) top view; (**b**) lateral view.

Before immersion in water, both sets of specimens were dried at a temperature of 40 °C for 8 h by using the precision composite curing oven of type OV301 manufactured by Easy Composites EU B.V. (Rijen, The Netherlands). The samples were weighed periodically until the mass stabilised, with an accuracy of 0.0001 g using DENVER SI-234A analytical balance (manufactured by Denver Instrument Co., Denver, CO, USA) with internal calibration, whose measuring range is 0–230 g. Then, the samples were immersed in water (Figure 4) and kept at room temperature (20 °C ± 2 °C) throughout the absorption curve study until saturation when the mass of the specimens had stabilised. As shown in Figure 4a, the samples were completely covered by water and placed on an aluminium support, which allows direct contact with water for all the surfaces of each specimen so that these specimens were not in contact with the walls or with the bottom of the vessel (Figure 4b). To maintain the immersion conditions, the water was refreshed weekly.

The immersion times were different for the two types of composite materials tested because the objective was to subject them to low-velocity impact tests after reaching saturation (when the absorbed moisture content tends to a constant value). Therefore, the total immersion time in the case of the samples made of CK composite material reinforced with eight layers of carbon–aramid hybrid fabric was approximately 11 months, while in the case of the sandwich samples made of CK2R composite material reinforced with carbon–aramid hybrid fabric and a rubber core was about 15 months (one year and three months). During the immersion period, the samples were weighed periodically using the same DENVER SI-234A analytical balance and the mass of the wet samples was recorded. The absorbed moisture of the samples expressed in percentage (%) was calculated according to the European standard EN ISO 62:2008 [55] by using Equation (1):

(1) $$m_{w}=\frac{m_{i}-m_{d}}{m_{d}}\cdot 100\left(\%\right),$$

where $m_{w}$—the mass of water absorbed by the specimen, expressed in percentages (%); $m_{d}$—the mass of the dried specimen (expressed in g); and $m_{i}$—the mass of the wet specimen after a certain period of immersion in water (expressed in g).

The absorption data obtained for all the samples manufactured of the same type of composite material were statistically processed. Finally, the average absorption curves were obtained for both the composite materials tested, reported to the square root of the immersion time.

Considering Fick’s second law of diffusion, the diffusion coefficient denoted with D is defined as the slope of the normalised mass uptake against the square root of immersion time $\sqrt{t}$ and is computed with Equation (2) [56,57]:

(2) $$D=\pi {\left(\frac{h}{4M_{m}}\right)}^{2}{tg}^{2}\alpha ,$$

where *h* is the thickness of the composite specimen immersed in water; $M_{m}$ is the equilibrium absorbed water inside the composite material at saturation, expressed in percentages; and tg α represents the initial slope of the curve of the absorbed moisture related to the square root of immersion time $\sqrt{t}$.

The initial slope tg α of the linear portion may be computed by using Equation (3) [58]:

(3) $$tg\alpha =\frac{m_{2}-m_{1}}{{\sqrt{t}}_{2}-{\sqrt{t}}_{1}}$$

where $\left(m_{1,}\;\sqrt{t_{1}}\right)\;$and $\left(m_{2,}\;\sqrt{t_{2}}\right)\;$are the coordinates of the two arbitrary points located on the linear portion of the plotted absorption curve.

#### 2.2.3. Post-Impact Microscopic Analysis

After the impact testing, we acquired photos of the damaged areas of the specimens by using the All-in-One HY-2070 digital optical microscope (manufactured by Shenzhen Hayear Electronics Co. Ltd., Shenzhen, China) in order to explain the failure modes in a low-velocity impact test. The digital microscope allows the standard magnification of 230× (maximum magnification is 500×) and the maximum image resolution may be 1920 × 1080. The microscope is digitally controlled by the Hayear software (version x64, 4.10.17214.20200601). We investigated the central and edge damaged areas in order to respond to some questions like the following: did break the both carbon and aramid fibres or just some fibres?; has delamination occurred at the fibre-matrix interface?

## 3. Results and Discussions

### 3.1. Results Obtained in Low-Velocity Impact Tests for Dried Specimens

**Figure 5:**
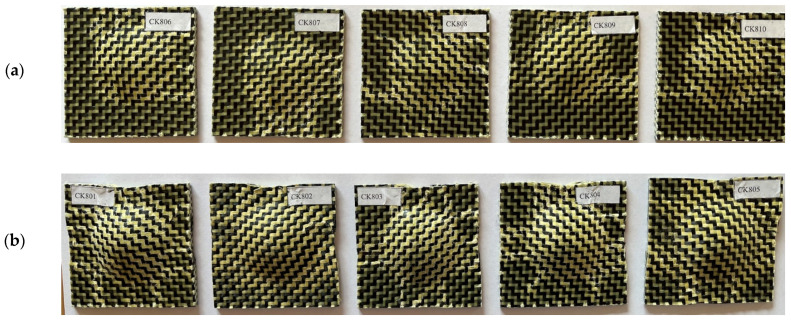
Photos of the plate composite specimens reinforced with eight layers of carbon–aramid hybrid fabric after the impact low-velocity tests: (**a**) for 25 J impact level; (**b**) for 50 J impact level.

In Figure 5, it can be observed that none of the CK composite specimens tested before immersion in water are not perforated in the low-velocity impact tests. The higher the impact energy, the larger the damaged area as a result of the increase in the energy absorbed for failure and plastic deformations.

After processing the experimental data, the results are synthesised in Table 4.

**Figure 6:**
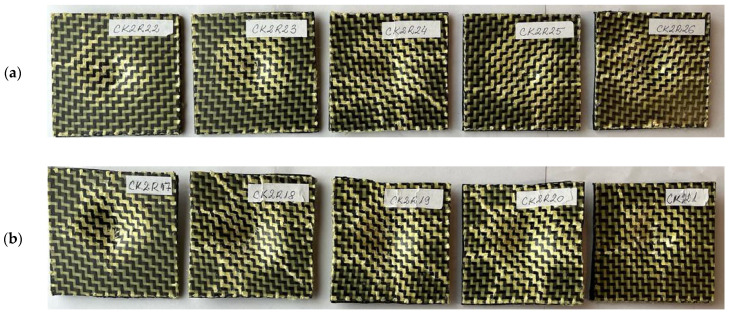
Photos of the sandwich hybrid composite specimens with a rubber core after the impact low-velocity tests at different impact energies: (**a**) 25 J; (**b**) 50 J.

In Figure 6, it can be observed that the impact samples manufactured by the sandwich composite material with a rubber core are not completely perforated after the low-velocity impact tests.

After processing the experimental data, the results are summarised in Table 5.

### 3.2. Comparative Analysis of the Impact Behaviour for Both Composite Materials Tested before Immersion in Water

**Figure 7:**
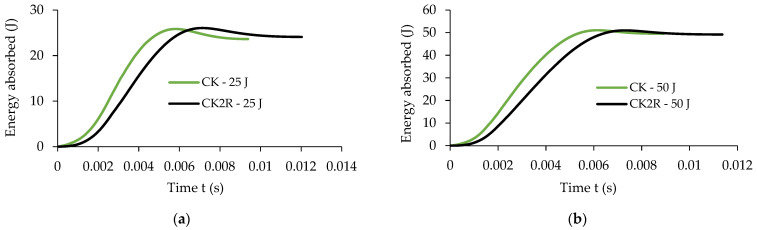

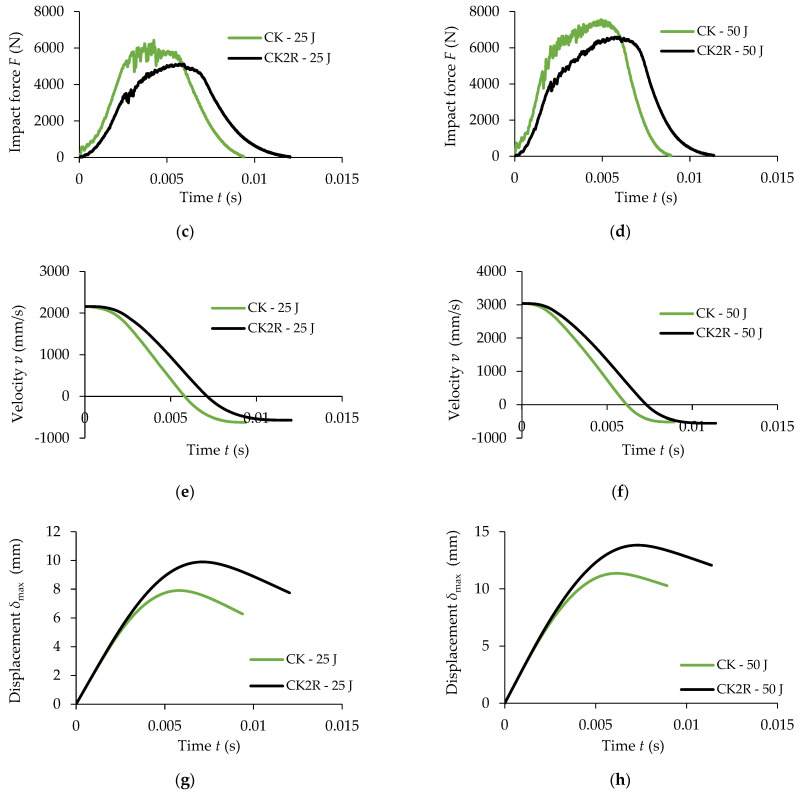
Comparative results recorded for the two sets of specimens in the low-velocity impact tests for the impact energy of 25 J and 50 J: (**a**,**b**) absorbed energy related to time; (**c**,**d**) impact force–time $\left(F-t\right)$; (**e**,**f**) velocity–time $\left(v-t\right)$; (**g**,**h**) displacement–time $\left(\delta_{max}-t\right)$.

Figure 7 presents comparative results obtained in the low-velocity impact tests for both types of specimens for both energy impact levels of 25 J and 50 J, respectively, regarding the following variation curves: the variation in the energy absorbed related to time, impact force–time $\left(F-t\right),$ velocity–time $\left(v-t\right),$ and displacement–time $\left(\delta_{max}-t\right)$.

A very important measured parameter during the low-velocity impact tests is the energy absorbed by the composite materials tested during impact tests. It is noted that in terms of the absorbed energy, there is a small difference between the two sets of samples; more specifically, for the impact energy of 25 J, the maximum energy absorbed by the sandwich sample CK2R with a rubber core is just 0.17% less than the energy absorbed by the sample CK without rubber core (Figure 7a). At the same time, for the impact energy of 50 J, the sandwich composite samples CK2R and the samples CK absorbed approximately the same quantity of energy, 49.77 J and 49.79 J, respectively (Figure 7b). However, it can be seen that at a certain moment of time, the energy absorbed by the impact plate CK is significantly higher than the energy absorbed by the plate CK2R having a sandwich composite structure. In conclusion, the replacement of the middle two layers reinforced with carbon–aramid hybrid fabric with the rubber core leads to a delay in energy absorption during impact while the absorbed energy is the same as the one absorbed by composite materials without a rubber core (Figure 7a,b).

The impact forces are greater for the CK composite materials than the ones recorded for the CK2R composite materials (Figure 7c,d). Regarding the time for which the velocity is equal to zero (Figure 7e,f), it can be noted that in the case of the dried CK specimens, for both impact energies, it is approximately equal to 6 ms. This time is approximately equal to 7 ms in the case of the CK2R specimens for both impact energies.

It can also be observed that in the case of the specimen CK2R, the maximum displacement $\delta_{max}$ of the impactor is about 26% and 22% higher for the impact energies of 25 J and 50 J, respectively, compared with the maximum displacement recorded for the composite specimen CK (Figure 7g,h).

### 3.3. Absorption Data

**Figure 8:**
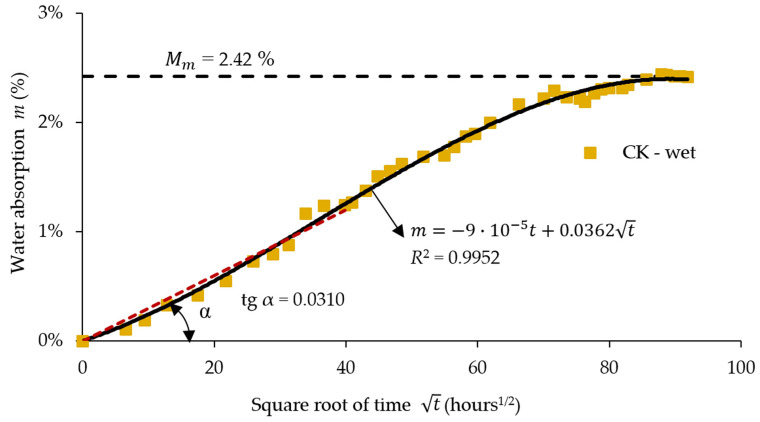
Absorption data during 8440 h of immersion in water ($R^{2}$ is a squared number for approximating the absorption data with the least squares method; red dot line represents the linear fuction which approximate the data of the absorption curve aacording to ISO 62:2008 [55]).

Figure 8 shows the moisture absorption curve during 8440 h of immersion in water based on the absorption data recorded for the aramid–carbon/epoxy resin hybrid composite material.

Analysing Figure 8, it may be noted that after approximately 8440 h of immersion in water, the absorption curve tends asymptotically to the equilibrium value $M_{m}$ which is equal to 2.42% for the composite specimens without rubber core.

**Figure 9:**
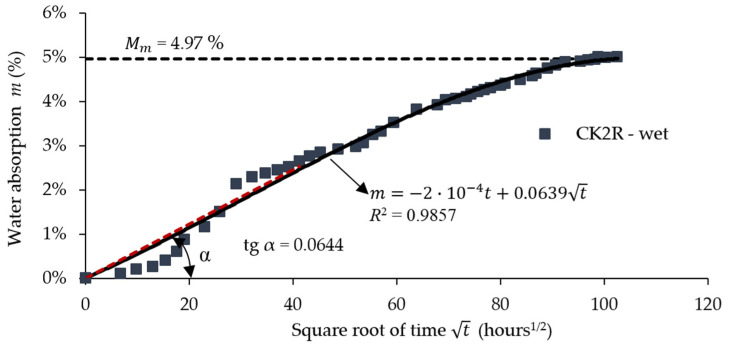
Absorption data during 10,513 h of immersion in water ($R^{2}$ is a squared number for approximating the absorption data with the least squares method; red dot line represents the linear fuction which approximate the data of the absorption curve aacording to ISO 62:2008 [55]).

Figure 9 shows the water absorption curve during 10,513 h of immersion in water for the aramid–carbon hybrid composite material having a rubber core. The absorbed water content tends to 4.97% after approximately 10,513 h of immersion in water for the specimens with rubber core (Figure 9). The initial portion of the absorption curve was approximated with a linear function graphically plotted as a red dot line (Figure 8 and Figure 9) by using the least squares method. According to Fick’s second law, the slope was equal to 0.0310 (Figure 8) for the specimens without a rubber core while the slope was equal to 0.0644 (Figure 9) for the specimens with a rubber core and these values were used to compute the diffusion coefficient *D* by using Equation (2).

Table 6 presents a resume of the calculated absorption data regarding the diffusion coefficient *D*, total immersion time, and moisture absorption at saturation. The moisture content *Mm* at the saturation of the CK composites involved in this study is approximately 48.70% lower than the corresponding value recorded in the case of the CK2R composites.

### 3.4. Results Obtained in Low-Velocity Impact Tests after Immersion in Water

After immersion in water and saturation is reached (when the absorbed moisture content tends to a constant value), the samples were subjected to the low-velocity impact test under the same test conditions as those presented in Section 2.2.1.

After processing the experimental data, the results are summarised in Table 7.

It may be noted that in the case of the impact testing of the wet CK composite plates after immersion for 8440 h in water (after saturation with water), the impact hammer ricochets with an average velocity of 0.56 m/s at an impact energy of 25 J, while the recoil does not occur at the impact energy of 50 J (Table 7).

**Figure 10:**
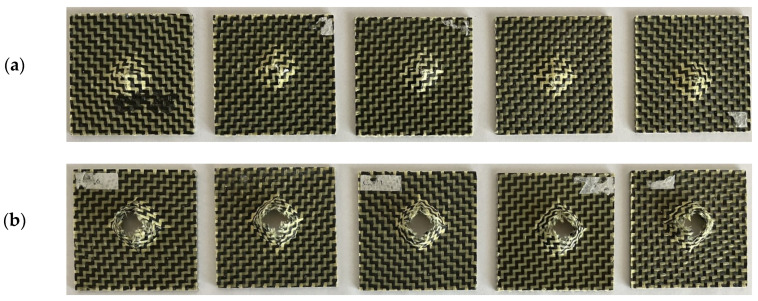
Photos of the CK specimens without a rubber core, subjected to low-velocity impact tests after 8440 h of immersion (after saturation) at different impact energies: (**a**) 25 J; (**b**) 50 J.

In Figure 10, it can be seen that wet CK composite samples without a rubber core are not completely perforated for the impact level of 25 J while all the wet samples were completely perforated for the impact level of 50 J. The average value of the absorbed energy for the complete breaking of all the layers (penetration) was 41.84 J (Table 7) for the CK composite materials after immersion in water for 8440 h.

The data reported in Table 8 regarding the average value of the absorbed energy show that less than 25 J is required to completely break all the layers of the wet CK2R sandwich composite specimens in the low-velocity impact tests after 10,513 h of immersion in water. The recoil does not occur in the low-velocity impact tests for any of the wet CK2R sandwich composite specimens tested (Table 8).

**Figure 11:**
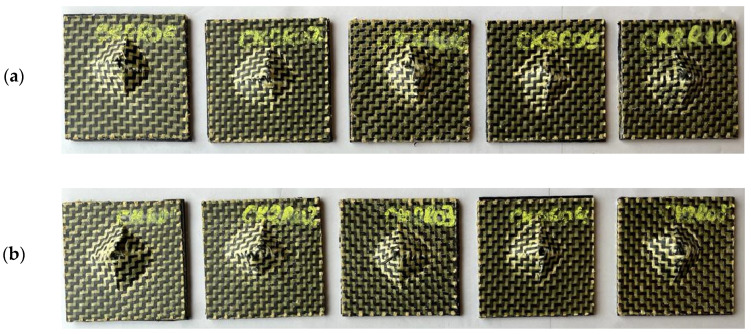
Photos of the CK2R sandwich composite specimens with a rubber core after the impact low-velocity tests performed after 10,513 h of immersion in water (after saturation) at both impact energies: (**a**) 25 J; (**b**) 50 J.

The photos shown in Figure 11 indicate that all the CK2R sandwich composite samples with a rubber core were fully perforated during the impact low-velocity tests carried out after immersion in water until saturation (10,513 h) in the case of both the impact energies.

### 3.5. Effects of Water Absorption on the Behaviour of the Composite Materials in Low-Velocity Impact Tests

**Figure 12:**
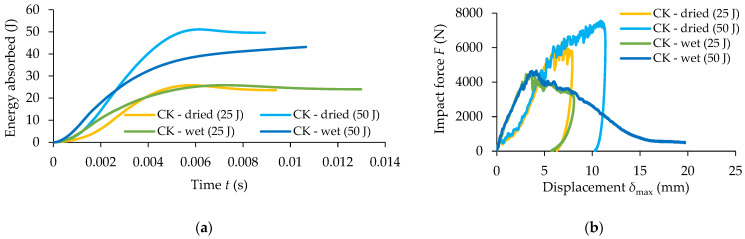

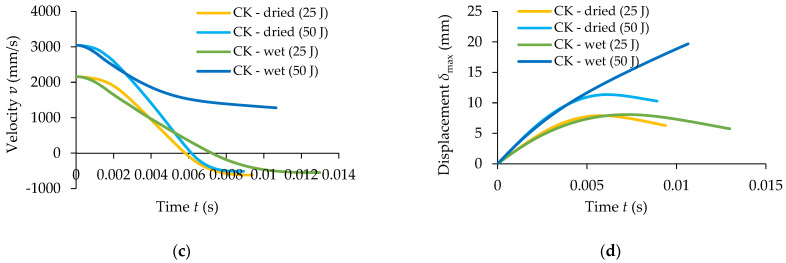
Comparative results obtained for wet and dried CK composite specimens for 25 J and 50 J impact energies: (**a**) absorbed energy related to time; (**b**) impact force–displacement $\left({F-\delta }_{max}\right)$; (**c**) velocity–time $\left(v-t\right);\;$(**d**) displacement–time $\left(\delta_{max}-t\right)$.

Figure 12 presents comparatively the results obtained for the dried and wet CK composite specimens in the low-velocity impact tests regarding the following curves: absorbed energy related to time, impact force–displacement $\left({F-\delta }_{max}\right)$, velocity–time $\left(v-t\right),$ and displacement–time $\left(\delta_{max}-t\right)$.

It can be noted from Figure 12a, that for 25 J impact energy, the absorbed energy is approximately the same for both CK dried specimens (23.73 J) and wet CK specimens (23.95 J). The difference is that more damages take place in the case of the wet CK specimens because all the layers are broken showing significant degradations of the carbon–aramid composite material, caused by the moisture absorption. However, for the CK specimens subjected to 50 J impact energy, a significant difference can be observed regarding both the evolution of the absorbed energy related to time and the value of the energy absorbed, as follows: the average value of the absorbed energy is 49.77 J for the dried specimens (see also Table 4) and it is equal with 41.84 J for the wet specimens (see also Table 7).

Analysing the curves from Figure 12b, it may be observed that the maximum force registered for the dried CK specimens is up to 67% and 39% higher than the ones recorded for the wet CK specimens for the same impact energy, for 50 J impact energy and for 25 J impact energy, respectively. Regarding the time when the velocity is equal to zero (Figure 12c) it can be noted that for the dried CK specimens, it is around 0.006 s for both the impact energies (see also Table 4), while for the CK wet specimens it is approximately 0.007 s for 25 J impact energy (see also Table 7). The maximum displacement of 7.97 mm (see also Table 4) recorded for the dried CK specimens for the 25 J impact energy is not significantly higher than the value of 7.95 mm (see also Table 7) recorded for the wet CK specimens. On the other hand, the average value of the maximum displacement recorded for 50 J impact energy in the case of testing of the dried CK specimens is 3.60 mm higher than the ones obtained for both dried and wet CK specimens subjected to 25 J impact energy (Figure 12d and see also Table 4 and Table 7).

**Figure 13:**
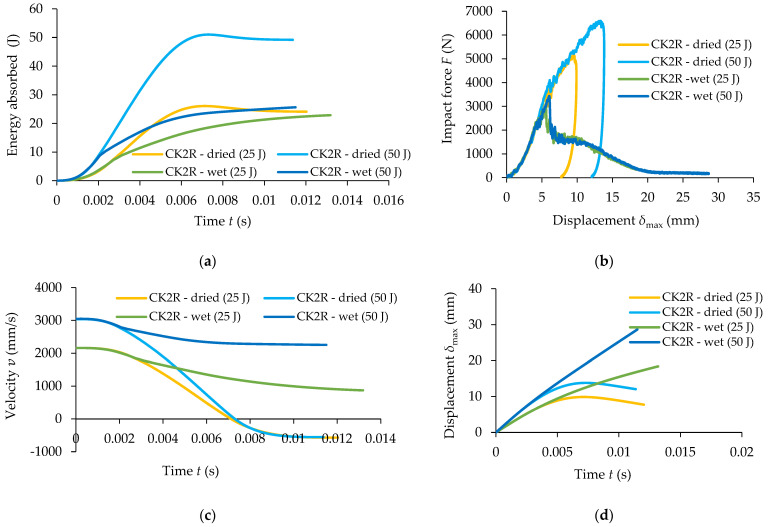
Comparative results between wet and dried CK2R composite specimens for 25 J impact level and 50 J impact level: (**a**) absorbed energy related to time; (**b**) impact forcedisplacement $\left({F-\delta }_{max}\right)$; (**c**) velocity–time $\left(v-t\right);$ (**d**) displacement–time $\left(\delta_{max}-t\right)$.

Figure 13 presents comparatively the results recorded for wet and dried CK2R composite specimens with rubber core, in the low-velocity impact tests, regarding the following variation curves: absorbed energy related to time, impact force–displacement $\left(F-\delta_{max}\right),$ velocity–time $\left(v-t\right),$ and displacement–time $\left(\delta_{max}-t\right)$.

The absorbed energy during the impact test is approximately the same for both the dried and wet CK2R specimens subjected to 25 J impact energy (Figure 13a). But, the evolution of the absorbed energy related to time is significantly different. However, for the CK2R specimens subjected to 50 J impact energy, a significant difference can be observed regarding the energy absorbed as follows: the average value of the absorbed energy is 49.77 J for dried CK2R specimens (see also Table 5) and the absorbed energy is 26.74 J for wet CK2R specimens (see also Table 8).

Analysing the curves shown in Figure 13b, it may be observed that the maximum force recorded for the dried CK2R specimens is 77% and 88% higher than the ones obtained for the wet CK2R specimens for 25 J impact energy and 50 J impact energy, respectively. Regarding the time for which the velocity is equal to zero (Figure 13c), it can be noted that for the dried CK2R specimens is approximately 7 ms for both impact energies (see also Table 5). On the other hand, the maximum displacement recorded for 50 J impact energy in the case of the testing of the dried CK2R specimens is 4.29 mm higher than the ones obtained for the specimens subjected to 25 J impact energy (Figure 13d).

### 3.6. Failure Modes in Low-Velocity Impact Test

**Figure 14:**
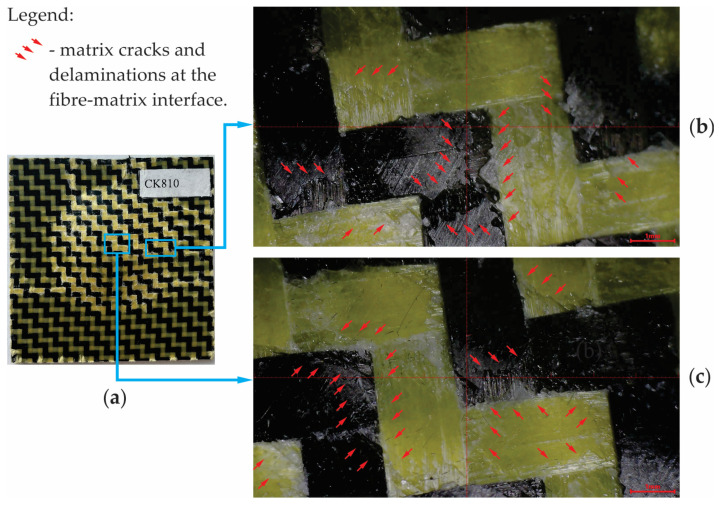
Failure modes for the dried CK specimens subjected to low-velocity impact at 25 J: (**a**) photo of the specimen after the impact test; (**b**) zoomed image (lens: ×100) of the areas located near the edge of the failure area; (**c**) zoomed image (lens: ×100) of the central areas of the failure area.

Photos of the central and edge areas of the specimens are acquired by using the All-in-One HY-2070 digital microscope after the impact test in order to investigate the failure modes. Just matrix cracks and delamination at the fibre–matrix interface develop in the case of the dried CK specimens subjected to the low-velocity impact test with an impact energy of 25 J (Figure 14).

**Figure 15:**
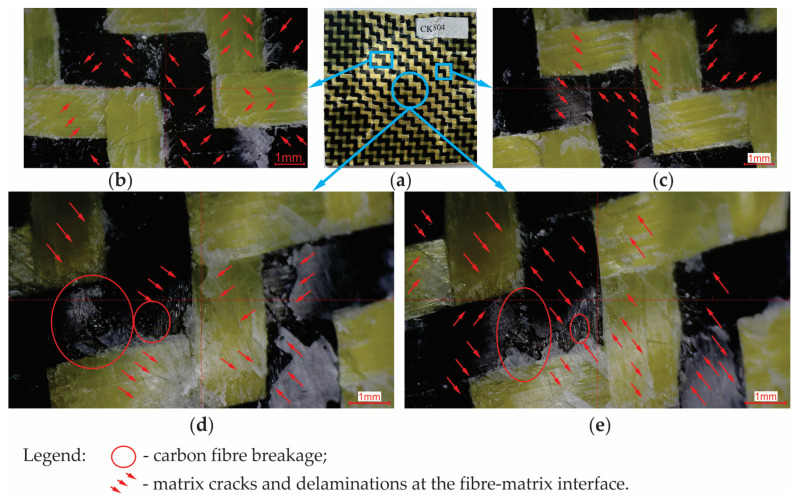
Failure modes for the dried CK specimens subjected to low-velocity impact at 50 J: (**a**) photo of the specimen after the impact test; (**b**,**c**) zoomed image of the areas located near the edge of the failure area; (**d**,**e**) zoomed image of the central areas of the failure area.

For the dried CK specimens subjected to the impact energy of 50 J, the breakage of the carbon fibre has been observed in addition to the matrix cracks and delamination of the fibre–matrix interface (Figure 15). It is noted that no breakage of the aramid fibres can be observed, and this is due to the fact that aramid fibres are more flexible than carbon ones, allow greater deformations, and store more strain energy until the rupture compared to the carbon fibres. For this reason, the aramid fibres are recommended for impact applications.

**Figure 16:**
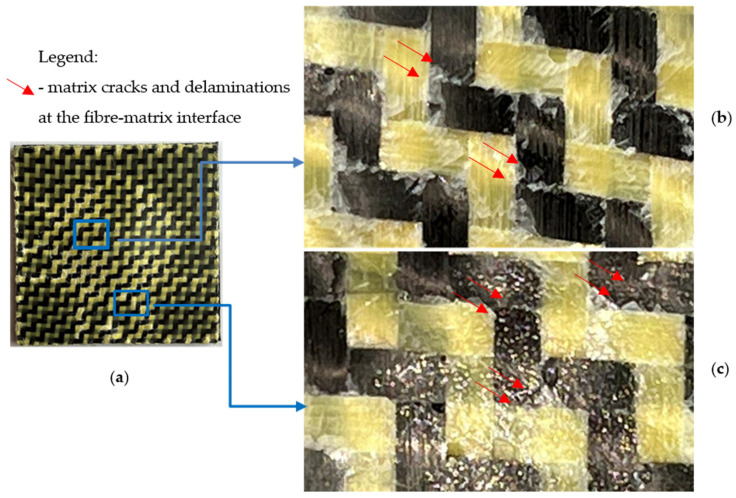
Failure modes for the dried CK2R specimens subjected to low-velocity impact at 25 J: (**a**) photo of the specimen after the impact test; (**b**) zoomed image (lens: ×100) of the central areas of the failure area; (**c**) zoomed image (lens: ×100) of the areas located near the edge of the failure area.

**Figure 17:**
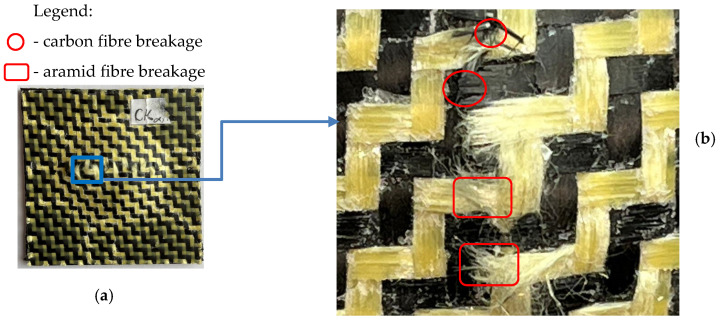
Failure modes for the dried CK2R specimens subjected to low-velocity impact at 50 J: (**a**) photo of the specimen after the impact test; (**b**) zoomed image (lens: ×100) of the central areas of the failure area.

In the case of the dried CK2R specimens subjected to 25 J impact energy, it can be observed similar post-impact failure modes as for the CK specimens, such as matrix cracks and delamination at the fibre–matrix interface (Figure 16). For the dried CK2R specimens subjected to 50 J impact energy, in addition, the breakages of both the carbon and aramid fibres can be observed (Figure 17).

Neither layer of the dried composite specimens is completely broken in low-velocity impact test. As a result, the impactor did not penetrate the hybrid dried composite plate specimen.

## 4. Conclusions

This work presented the experimental results obtained for two types of plates made of carbon–aramid composite materials in low-velocity drop tests for two levels of the impact energies of 25 J and 50 J, respectively. The main contributions of this research concern both the comparison of the behaviour of the two different carbon–aramid composite materials involved in this study (with and without a rubber core) and the investigation of the effects of the water absorption on the impact behaviour and on the failure modes. Finally, the main conclusions of this research are synthesised below.

- Maximum displacement $\delta_{max}$ recorded for the CK2R sandwich composites with a rubber core is 26% and 22% higher for the impact energies of 25 J and 50 J, respectively, than the values recorded for the CK carbon–aramid composite materials. It is obvious that the time until reaching the maximum displacement is greater for the CK2R sandwich composites with a rubber core.
- The replacement of the middle two layers reinforced with carbon–aramid hybrid fabric with the rubber core in the sandwich composite leads to a delay in energy absorption during the low-velocity impact test while the absorbed energy is approximately the same as the one absorbed by the CK composite materials at the final of the impact test for both the 25 J and 50 J impact energies.
- In the case of impact tests for the dry specimens, matrix cracks and delamination at the fibre–matrix interface developed in the low-velocity impact test at an impact energy of 25. It was found that at an impact energy of 50 J, both the carbon fibres and aramid fibres break in the sandwich composite materials with rubber core, while only breakages of carbon fibres are observed in the composite material without rubber core. A complete rupture of all layers was not observed in the case of both composite materials;
- For the CK2R sandwich composite materials with rubber core, the water content of 4.97% at saturation is twice the water content (2.42%) absorbed by the CK composite materials until saturation.
- In the case of testing of the CK composite materials reinforced with carbon–aramid fabrics, with the impact energy of 50 J, the average value of the absorbed energy was 41.84 J for the complete breakage of all the layers after 8440 h of immersion in water while the absorbed energy was 49.79 J for dried specimens which were not perforated. It follows that the absorbed energy of the wet CK specimens was approximately 16% less than the one absorbed by dried specimens. The damages occurred for the CK dried specimens are carbon fibre breakage, matrix cracks, and delamination at matrix-fibre interfaces.
- In the case of testing of the CK2R composite materials, the average value of the absorbed energy was 23.26 J for the complete breakage of all the layers after 10,513 h of immersion in water while the absorbed energy was 49.77 J for dried specimens which were not perforated. Therefore, the decrease in the absorbed impact energy was 53.26% after 10,513 h of immersion in water. The damages that occurred for the CK2R dried specimens are breakage of both the carbon and aramid fibres, matrix cracks, and delamination at matrix–fibre interfaces.
- The replacement of two core layers reinforced with carbon–aramid fabric with the rubber core is a reliable solution for the applications of these types of composite materials in dried environments because the degradation of the impact properties is much more pronounced for the composite material with rubber core after water absorption.

The findings of this research should be taken into account in designing of parts made of composite materials reinforced with carbon–aramid fabrics, used for impact protection in applications that works in wet environments.

## Figures and Tables

**Table 1:** Characteristics of the carbon–aramid hybrid woven fabric used for reinforcement [48].

Reinforcement Material	Thread Counts per cm (Warp/Weft Direction)	Type of Woven Fabric	Density per Area Unit (g/m^2^)	Fineness of Yarns (dtex)
SIGRATEX H W215-TW2/2	5.7	Twill 2/2	215	2000 (for C ^1^) 1600 (for K ^1^)

^1^ C—carbon yarn; K—aramid yarn.

**Table 2:** Properties of EPOLAM 2031 epoxy resin complex [49].

Material	Tensile Modulus (MPa)	Tensile Strength (MPa)	Flexural Modulus *E* (MPa)	Flexural Strength (MPa)
EPOLAM 2031 epoxy resin complex	3600	80	2900	130

**Table 3:** Material structure and number of specimens for the low-velocity impact testing.

Composite Material	Specimen Codes ^1^	Material Structure of the Specimen	Type of Specimens	Number of Specimens Tested	
				Impact Energy 25 J	Impact Energy 50 J
Carbon–aramid/epoxy Epolam 2031	CK801…CK810	Panel having eight layers reinforced with carbon–aramid hybrid fabric	Dried specimens	5	5
	CK811…CK815, CK818…CK822		Wet specimens	5	5
Carbon–aramid with rubber core/epoxy Epolam 2031	CK2R17…CK2R26	Sandwich panel with both faces made of composite material containing three layers reinforced with carbon–aramid hybrid fabric and a rubber core	Dried specimens	5	5
	CK2R01…CK2R10		Wet specimens	5	5

^1^ CK8XX: CK8—eight layers of aramid–carbon fabric; XX—specimen number; CK2RXX: CK—faces reinforced with aramid–carbon fabric; 2R—rubber core with thickness of 2 mm; XX—number.

**Table 4:** Results obtained in the low-velocity impact tests for the impact-dried CK composite specimens reinforced with eight layers of carbon–aramid hybrid woven fabric.

Impact Energy *E* (J)	Specimen Code ^1^	Thickness of the Specimen *h* (mm)	Absorbed Energy *W* (J)	Maximum Displacement *δ*_max_ at Velocity *v = 0* (mm)	Time *t* at Maximum Displacement *δ*_max_ (s)	Recoil Velocity *v*_R_ (m/s)
25	CK806	2.61	23.79	7.96	0.0058	−0.60
	CK807	2.55	23.57	7.91	0.0057	−0.63
	CK808	2.65	23.63	7.91	0.0058	−0.62
	CK809	2.59	23.94	8.19	0.0061	−0.58
	CK810	2.62	23.74	7.86	0.0058	−0.61
	Average (stdev)	2.60 (0.037)	23.73 (0.144)	7.97 (0.130)	0.0058 (0.00015)	−0.61 (0.019)
50	CK801	2.62	49.91	11.69	0.0064	−0.46
	CK802	2.65	49.87	11.63	0.0063	−0.46
	CK803	2.61	49.97	11.63	0.0064	−0.45
	CK804	2.61	49.57	11.36	0.0062	−0.51
	CK805	2.62	49.63	11.63	0.0062	−0.51
	Average (stdev)	2.62 (0.016)	49.79 (0.178)	11.59 (0.130)	0.0063 (0.0001)	−0.48 (0.030)

^1^ CK8XX: CK8—eight layers of aramid–carbon hybrid fabric; XX—specimen number.

**Table 5:** Results obtained in the low-velocity impact tests for the CK2R sandwich hybrid composite dried specimens having rubber core.

Impact Energy *E* (J)	Specimen Code ^1^	Thickness of the Specimen *h* (mm)	Absorbed Energy *W* (J)	Maximum Displacement *δ*_max_ at Velocity *v* = 0 (mm)	Time *t* at Maximum Displacement *δ*_max_ (s)	Recoil Velocity $v_{R}$ (m/s)
25	CK2R22	3.74	23.91	10.41	0.0077	−0.61
	CK2R23	3.83	23.92	9.65	0.0069	−0.56
	CK2R24	3.82	23.37	9.33	0.0068	−0.63
	CK2R25	3.74	24.11	9.90	0.0079	−0.57
	CK2R26	3.85	23.16	9.87	0.0071	−0.67
Average (stdev)	- -	3.79 (0.052)	23.69 (0.406)	9.83 (0.40)	0.00728 (0.000492)	−0.61 (0.045)
50	CK2R17	3.74	49.97	14.22	0.0082	−0.47
	CK2R18	3.72	49.14	13.55	0.0071	−0.61
	CK2R19	3.70	49.21	13.82	0.0073	−0.55
	CK2R20	3.76	49.82	13.88	0.0073	−0.51
	CK2R21	3.83	50.69	15.13	0.0099	−0.36
Average (stdev)	- -	3.75 (0.050)	49.77 (0.632)	14.12 (0.613)	0.00796 (0.001165)	−0.50 (0.094)

^1^ CK2RXX code: C—carbon; K—aramid; 2R—rubber core 2 mm; XX—number of specimens.

**Table 6:** Absorption data for the composite materials immersed in water.

Specimen Code ^1^	Total Immersion Time at Saturation (hours)	Moisture Content at Saturation *M*_m_ (%)	Diffusion Coefficient *D* (10^−6^ mm^2^/s)
CK	8440	2.42	0.0605
CK2R	10,513	4.97	0.1287

^1^ CK code: C—carbon; K—aramid; CK2R code: C—carbon; K—aramid; 2R—rubber core 2 mm.

**Table 7:** Results obtained in the low-velocity impact tests for the wet CK composite specimens immersed for 8440 h (after saturation) reinforced with eight layers of carbon–aramid hybrid fabric.

Impact Energy *E* (J)	Specimen Code	Thickness of the Specimen *h* (mm)	Absorbed Energy *W* (J)	Maximum Displacement *δ*_max_ at Velocity *v* = 0 (mm)	Time *t* at Maximum Displacement *δ*_max_ (s)	Recoil Velocity $v_{R}$ (m/s)
25	CK811	2.61	24.01	8.08	0.0071	−0.55
	CK812	2.62	23.86	7.91	0.0069	−0.57
	CK813	2.59	24.02	7.87	0.0069	−0.55
	CK814	2.60	24.02	8.08	0.0072	−0.55
	CK815	2.58	23.86	7.80	0.0068	−0.57
	Average (stdev)	2.60 (0.0158)	23.95 (0.086)	7.95 (0.127)	0.00698 (0.000164)	−0.56 (0.011)
50	CK818	2.63	42.74	-	-	-
	CK819	2.62	44.33	-	-	-
	CK820	2.60	43.17	-	-	-
	CK821	2.59	40.43	-	-	-
	CK822	2.58	38.51	-	-	-
	Average (stdev)	2.60 (0.0207)	41.84 (2.338)	-	-	-

**Table 8:** Results obtained in low-velocity impact tests carried out on CK2R sandwich composite specimens with rubber core after 10,513 h of immersion in water (after saturation).

Impact Energy *E* (J)	Specimen Code ^1^	Thickness of the Specimen *h* (mm)	Absorbed Energy *W* (J)	Maximum Displacement *δ*_max_ at Velocity *v* = const. (mm)	Time *t* at Maximum Displacement *δ*_max_ at Velocity *v* = const. (s)	Recoil Velocity $v_{R}$ (m/s)
25	CK2R6	3.77	24.03	17.63	0.0133	-
	CK2R7	3.69	23.00	18.22	0.0131	-
	CK2R8	3.77	23.33	17.55	0.0128	-
	CK2R9	3.60	22.88	18.42	0.0132	-
	CK2R10	3.63	23.07	18.59	0.0132	-
	Average (stdev)	3.69 (0.0782)	23.26 (0.460)	18.08 (0.469)	0.01312 (0.00019)	-
50	CK2R1	3.70	27.48	30.01	0.0113	-
	CK2R2	3.74	23.58	18.96	0.0073	-
	CK2R3	3.65	24.19	21.42	0.0083	-
	CK2R4	3.76	26.81	36.18	0.0148	-
	CK2R5	3.65	31.66	29.42	0.0127	-
	Average (stdev)	3.70 (0.0505)	26.74 (3.211)	27.20 (6.978)	0.01088 (0.0031)	-

^1^ CK2RXX: C—carbon; K—aramid; 2R—rubber core 2 mm; XX—number of specimens.

## Data Availability

Data are contained within the article.
